# Acetylation, Methylation and Allysine Modification Profile of Viral and Host Proteins during Influenza A Virus Infection

**DOI:** 10.3390/v13071415

**Published:** 2021-07-20

**Authors:** Farjana Ahmed, Torsten Kleffmann, Matloob Husain

**Affiliations:** 1 Department of Microbiology and Immunology, University of Otago, P.O. Box 56, Dunedin 9054, New Zealand; farjana.ahmed@postgrad.otago.ac.nz; 2 Centre for Protein Research, Research Infrastructure Centre, University of Otago, P.O. Box 56, Dunedin 9054, New Zealand; torsten.kleffmann@otago.ac.nz

**Keywords:** influenza A virus, proteomic, protein modifications, methylation, acetylation, allysine

## Abstract

Protein modifications dynamically occur and regulate biological processes in all organisms. Towards understanding the significance of protein modifications in influenza virus infection, we performed a global mass spectrometry screen followed by bioinformatics analyses of acetylation, methylation and allysine modification in human lung epithelial cells in response to influenza A virus infection. We discovered 8 out of 10 major viral proteins and 245 out of 2280 host proteins detected to be differentially modified by three modifications in infected cells. Some of the identified proteins were modified on multiple amino acids residues and by more than one modification; the latter occurred either on different or same residues. Most of the modified residues in viral proteins were conserved across >40 subtypes of influenza A virus, and influenza B or C viruses and located on the protein surface. Importantly, many of those residues have already been determined to be critical for the influenza A virus. Similarly, many modified residues in host proteins were conserved across influenza A virus hosts like humans, birds, and pigs. Finally, host proteins undergoing the three modifications clustered in common functional networks of metabolic, cytoskeletal, and RNA processes, all of which are known to be exploited by the influenza A virus.

## 1. Introduction

The influenza virus is a globally prevalent human respiratory pathogen, with recurring epidemic and pandemic potentials. Practically, the influenza A virus (IAV) will be impossible to eradicate because of its intrinsic evolving nature, zoonotic potential, and broad host range which includes migratory birds. Hence, there will be a constant need to develop new vaccines and antiviral drugs. Like all viruses, IAV is an obligate intracellular pathogen and exploits the host machinery to multiply and cause flu [1]. IAV utilises all three parts: the plasma membrane, cytoplasm, and nucleus, of the host cell to complete its life cycle. For this, IAV employs viral proteins and exploits host proteins to execute multiple crucial functions, such as anterograde and retrograde intracellular trafficking, RNA processing, and antagonism of host antiviral response. Many of these functions involve numerous protein-protein, protein-nucleic acid, and protein-lipid interactions. Evidently, these interactions are known to be regulated by multiple protein modifications, which occur co- or post-translationally and can be reversible and irreversible. Further, the dysregulation or imbalance of protein modifications is a hallmark of various human diseases [2,3]. Therefore, it is conceivable that the modifications of host proteins that are involved in IAV infection play an important role in their function. Furthermore, the magnitude of the modifications of such proteins is expected to change in response to IAV infection and, in addition to host proteins, IAV proteins also are expected to undergo those modifications.

Indeed, the modifications like phosphorylation, glycosylation, and ubiquitination have been described to differentially occur on both viral and host proteins and be significant during IAV infection [4,5]. However, the occurrence and role of other modifications such as acetylation, methylation and allysine modification during IAV infection are poorly understood. Acetylation is the addition of an acetyl group to lysine or serine side chains or the N-terminal amino group of proteins [2,6], whereas methylation is the addition of a methyl group to lysine or arginine side chains [7]. Both lysine methylation and acetylation are reversible modifications, and methylation can influence the acetylation or other modifications of the same or a neighbouring residue [8]. Allysine (α-aminoadipic-delta-semialdehyde) is the oxidative deamination of lysine, hydroxylysine, methyl-lysine or acetyl-lysine side chains and catalysed by lysyl oxidase [9,10,11]. Allysine can form inter- and intra-molecular cross-linkages with other allysine or lysine residues [9,12,13].

Recently, we described the antiviral role of multiple class I, II and IV host deacetylases during IAV infection [14,15,16,17,18]. Further, Koyuncu et al. described a similar role for class III deacetylases (known as sirtuins) in IAV infection [19]. These findings indicated that the host acetylation machinery plays a pro-viral role during IAV infection and, potentially, both viral and host proteins are differentially modified by acetylation to facilitate this. Indeed, recent reports have demonstrated that IAV nucleoprotein (NP) undergoes acetylation [20,21], and the activities of IAV RNA polymerase acidic (PA) protein [22] and host shut-off protein, PA-X [23] are promoted by acetylation. Furthermore, IAV PA, RNA polymerase basic 1 (PB1) protein, matrix protein M1 and the non-structural proteins, NS1 and NS2 have been shown to be acetylated at the N-terminus [24]. In addition, the acetylation of host tubulin [25] and histone 3 [17] is augmented during IAV infection. However, the role of methylation during IAV infection has been described sparingly [4,26] and we have not come across a report describing the role of allysine during IAV infection. Evidently, both methylation and acetylation, and potentially allysine can occur interchangeably on the same lysine residue [27,28]. Furthermore, there is evidence of functional crosstalk between acetylation and methylation and other protein modifications [8,29]. Hence, it is conceivable that in addition to acetylation, both viral and host proteins are modified by different lysine modifications such as methylation and allysine during IAV infection.

To identify a repertoire of both viral and host proteins that are differentially modified by acetylation, methylation and allysine during IAV infection, we employed a global profiling approach by liquid chromatography-coupled tandem mass spectrometry (LC-MS/MS) followed by functional annotation of modified proteins using various bioinformatics tools. Here, we report that many viral and host proteins are indeed modified on multiple amino acid residues by three modifications during IAV infection. Furthermore, most of the modified residues are conserved and critical for the function of those viral and host proteins. Finally, many of the identified host proteins undergoing modifications are already known to play a critical role in IAV infection, indicating the significance of these modifications.

## 2. Materials and Methods

### 2.1. Cells, Viruses, and Infection

Human lung alveolar epithelial cells, A549 were grown and maintained in minimum essential medium (MEM) supplemented with 10% foetal bovine serum, 1% L-glutamine and 1% penicillin-streptomycin (Life Technologies, Carlsbad, CA, USA) under 5% CO_2_ atmosphere and at 37 °C. Influenza virus A/Puerto Rico/8/1934 (H1N1) was propagated in 10 days old embryonated chicken eggs and titrated in Madin Darby canine kidney (MDCK) cells. For infection, confluent A549 cell monolayers, prewashed twice with serum-free MEM, were incubated with virus inoculum (multiplicity of infection of 1.0) in serum-free MEM for 1 h at 35 °C. The inoculum was removed, cells were washed once with serum-free MEM, replenished with fresh serum-free MEM, and incubated at 35 °C. After 24 h, the culture medium and the cells were harvested separately. The medium was titrated to measure the amount of released viral progeny and confirm a productive infection.

### 2.2. In-Gel Digestion

Cells were lysed in a lysis buffer (50 mM Tris-HCl pH 7.4, 150 mM NaCl, 1% Triton X-100, 0.5% sodium deoxycholate, 0.5% sodium dodecyl sulfate, and 1X protease inhibitor cocktail). The protein amount per sample was quantified using the Pierce™ BCA protein assay kit (ThermoFisher Scientific, San Jose, CA, USA). Equal amounts of protein were loaded and resolved on 15% SDS-PAGE along with SeeBluePlus 2 pre-stained molecular weight standards (Life Technologies, Carlsbad, CA, USA). Proteins were prefixed in-gel with 50% methanol and 10% glacial acetic acid in water, then stained with Coomassie Blue R-250 solution (Sigma-Aldrich, St Louis, MO, USA) for 1 h and de-stained overnight with 40% methanol and 10% glacial acetic acid in water. An image of the gel was acquired on an Odyssey Fc imaging system using Image Studio software version 5.0 (Li-COR, Lincoln, NE, USA). Each gel lane was cut into 10 molecular weight fractions and proteins were in-gel digested with trypsin using an automated digestion procedure (Intavis AG, Koln, Germany) described elsewhere [30]. Eluates containing the peptides were dried by centrifugal vacuum concentration.

### 2.3. Filter-Aided Sample Preparation (FASP)

Cells were lysed in a lysis buffer containing 40 mM Tris base, 1 mM EDTA, 0.5 M TCEP, 0.5% SDS, 0.5% sodium deoxycholate, 0.1% NP-40 and 1X protease inhibitor cocktail. The lysates were centrifuged at 16,000× *g* for 30 min and the supernatants were further processed by the FASP method described elsewhere [31]. The resulting protein amount was quantified by Bradford assay and digested with trypsin overnight. Peptides were dried and concentrated as above.

### 2.4. Mass Spectrometry Analysis

The dried peptides were resolubilized in 5% acetonitrile (ACN) and 0.1% formic acid (FA) in water and analysed using the Ultimate 3000 nano-flow uHPLC-System (Dionex Co, San Jose, CA, USA) in-line coupled to the nanospray source of LTQ-Orbitrap XL mass spectrometer (ThermoFisher Scientific, San Jose, CA, USA). The peptides were separated on in-house packed emitter tip columns (75 μm ID fused silica tubing filled with C-18 material to a length of 12 cm) at a flow rate of 400 nL/min. The liquid chromatography gradient gradually increased from 5% (*v/v*) ACN, 0.1% (*v/v*) FA to 45% ACN, 0.1% (*v/v*) FA and then to 95% (*v/v*) ACN, 0.1% (*v/v*) FA. Full mass spectra were acquired in the Orbitrap analyser in a mass range between *m/z* 400–2000 and a mass resolution of 60,000 at *m/z* 400. The five strongest signals were selected for collision-induced dissociation-MS2 acquisition in the LTQ ion trap at a normalised collision energy of 35%. At least two technical replicates of each of the biological replicates were analysed.

### 2.5. Data Analysis

Raw data were pooled from three independent biological replicates and was analysed using the Proteome Discoverer software (version 2.2, ThermoFisher Scientific, San Jose, CA, USA) and searched against a customised database containing human and influenza virus A/Puerto Rico/8/1934 (H1N1) reference amino acid sequences (55,836 entries downloaded on 23 June 2018 from the NCBI server, https://www.ncbi.nlm.nih.gov/) using the SEQUEST HT search engine (ThermoFisher Scientific, San Jose, CA, USA). The search was set up for full tryptic peptides with a maximum of 3 missed cleavages. The precursor mass tolerance threshold was fixed at 10 ppm with a maximum fragment mass error of 0.8 Da. Carbamidomethyl (C) was set as a static modification and acetylation (K, S), lysine > allysine (K), methylation (K, R) as dynamic peptide modifications. N-terminal acetylation with the dynamic loss of the N-terminal methionine was set as a dynamic protein modification. Proteins were considered identified only when at least two significant peptide hits were detected. Peptide hits were considered significant when passing the adjusted score threshold at a strict False Discovery Rate (FDR) of q < 0.01 as calculated by the percolator algorithm [32]. Protein modifications were considered only if a given modification in a peptide was identified in at least two biological replicates.

### 2.6. Bioinformatics Analysis

All protein sequences analysed here were extracted from NCBI databases and aligned by BioEdit (version 7.2.5) using ClustalW multiple sequence alignments. The conservation of amino acid residues was visualised using WebLogo (version 2.8.2). The 3-dimensional (3D) structure of viral proteins, acquired from the RCSB protein data bank (PDB) was visualized and analysed by PyMOL (version 2.3.4, Schrödinger LLC, New York, NY, USA). The acetylation site prediction was performed using ASEB web software (http://bioinfo.bjmu.edu.cn/huac/predict_p/, accessed on 28 February 2020). The STRING database (version 11.0) was used to generate functional association networks of modified host proteins at a confidence score of ≥ 0.7 (high confidence). The resultant networks were further analysed by Cytoscape (version 3.8.0) for densely connected regions using the Molecular Complex Detection (MCODE) algorithm. The four highest-ranked clusters were selected as the most significant clusters.

## 3. Results

Human lung alveolar epithelial cells, A549 were either mock-infected or infected with influenza virus A/Puerto Rico/8/1934 (H1N1) strain at the multiplicity of infection (MOI) of 1.0. After 24 h, the culture medium and the cells were harvested separately. The medium was titrated to detect viral progeny and confirm productive infection (Supplementary Figure S1A). The cells were lysed, and the lysates were processed to prepare LC-MS/MS samples using either in-gel (Supplementary Figure S1B) or in-solution digestion procedures (Supplementary Figure S1C). The raw data obtained from three independent biological replicates were pooled and searched against a customised database comprising 55,836 influenza virus A/Puerto Rico/8/1934 (H1N1) and human reference sequences using Proteome Discoverer software and SEQUEST HT search engine with appropriate protein modification filters.

### 3.1. Eight Viral Proteins Were Modified by Acetylation, Methylation and/or Allysine

IAV genome encodes 10 major viral proteins and at least 7 accessory viral proteins [33]. We detected all 10 major IAV proteins: hemagglutinin (HA), matrix proteins (M1 and M2), nucleoprotein (NP), neuraminidase (NA), NS1 and NS2, and RNA polymerase subunits PA, PB1, and PB2 with a sequence coverage ranging from 89% to 30% (Supplementary Table S1). Out of these, eight proteins: HA, M1, NP, NS1, NS2, PA, PB1, and PB2 were found to be modified by acetylation, methylation or allysine (Table 1). In some viral proteins, individual modifications occurred on multiple amino acid residues. Furthermore, most of the viral proteins were modified by at least two modifications on different residues, but the same residues were also detected to be alternatively modified by different modifications (Table 1).

**Figure 1:**
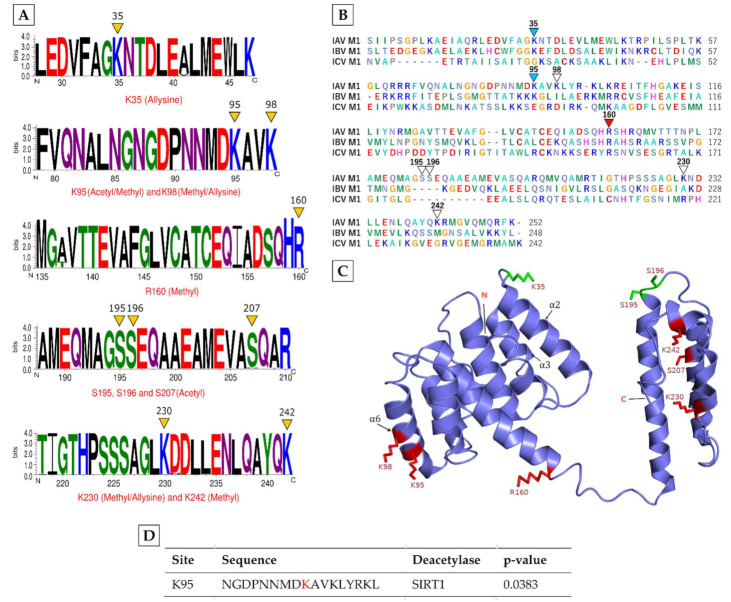
The characteristics of the modifications on IAV M1 protein. (**A**) Conservation of modified residues across M1 of IAV subtypes. A total of 800 sequences from 39 different IAV subtype strains were aligned in BioEdit using ClustalW and visualised by WebLogo. The logos represent the individual peptides with modified residues (yellow arrowheads). The *X*-axis indicates the position of amino acid and the *Y*-axis indicates the bit score on a scale of 1–4 (A scale of 4 means 100% conservation). Acetyl: acetylation, Methyl: methylation. (**B**) Conservation of modified residues across M1 of IAV, influenza B virus (IBV), and influenza C virus (ICV). The M1 sequences from IAV, IBV, and ICV reference sequences were aligned as above. Red arrowheads point to the conservation of modified residue in all three influenza virus types, turquoise arrowheads point to conservation in IAV and IBV, and white arrowheads point to no conservation. (**C**) Location of modified residues in the M1 structure. Red atom stick indicates the location on α-helix regions, green atom stick indicates the location on loop regions. N, N-terminal domain; C, C-terminal domain. (**D**) Prediction of identified acetylated sites (in red) to be acetylated or deacetylated by ASEB web software.

**M1**, which forms a shell under the envelope of IAV particles and plays critical roles during IAV assembly, was found to be modified by all three modifications, i.e., methylation, acetylation and allysine (Table 1). Methylation occurred on lysine (K) 95, K98, arginine (R) 160, K230, and K242; whereas acetylation was detected on serine (S) 195, S196, and S207 and K95; and allysine was found on K35, K98, and K230 (Table 1, in red). Interestingly, three residues: K95, K98 and K230 were found to be differentially modified by either methylation, acetylation or allysine (Table 1, asterisks). To understand the significance of these modifications, we aligned at least 800 M1 sequences from 39 different IAV subtypes, ranging from H1N1 to H13N6 (Supplementary Table S2), and found that, except S207, all identified modified residues in M1 were highly conserved across IAV subtypes (Figure 1A). Furthermore, R160 was also conserved in M1 of all three main types of influenza viruses, i.e., IAV, influenza B virus (IBV) and influenza C virus (ICV); whereas K35 and K95 were conserved within M1 of IAV and IBV (Figure 1B). Next, we determined the location of modified residues in the M1 structure, which comprises 12 α-helices and several loop regions but no β-strands [34,35]. The allysine K35 was located on the loop between α2 and α3 helices, whereas the acetylated S195 and S196 were located on the loop between α10 and α11 helices (Figure 1C). The rest of the modified residues were located on different α-helices (Figure 1C). Particularly, K95 and K98 were located on “helix six”, which is critical for various M1 functions, including in IAV assembly and particle shape and integrity [36]. Finally, we used Acetylation Set Enrichment Based (ASEB) web software [37] to predict whether some of the identified acetylated sites in M1 can be acetylated or deacetylated by host lysine acetyltransferases (KAT) or deacetylases, respectively. The ASEB analysis revealed that acetylation at K95 can be deacetylated by sirtuin-1 (SIRT1) deacetylase (Figure 1D).

**Figure 2:**
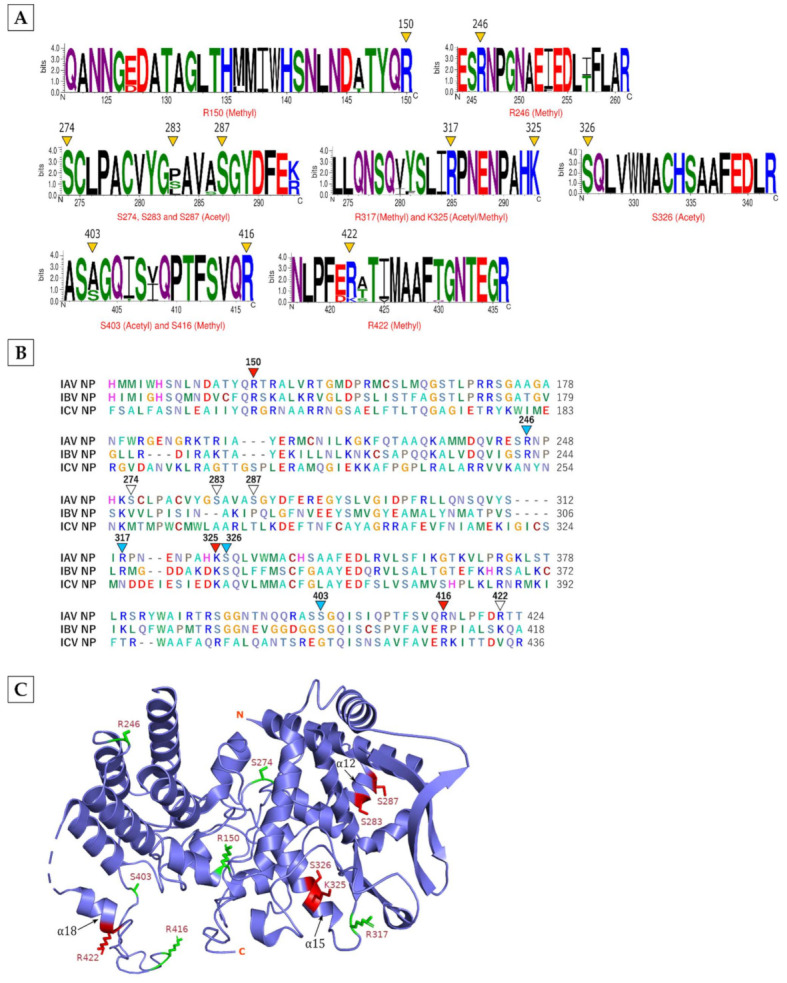
The characteristics of the modifications on IAV nucleoprotein (NP). (**A**) Conservation of modified residues across NP of IAV subtypes. A total of 1,124 sequences from 42 different IAV subtype strains were aligned in BioEdit using ClustalW and visualised by WebLogo. The logos represent individual peptides with modified residues (yellow arrowheads). The *X*-axis indicates the position of amino acid and the *Y*-axis indicates the bit score on a scale of 1–4 (A scale of 4 means 100% conservation). Acetyl: acetylation, Methyl: methylation. (**B**) Conservation of modified residues across NP of IAV, IBV, and ICV. Red arrowheads point to conservation in all three influenza virus types, turquoise arrowheads point to conservation in IAV and IBV, and white arrowheads point to no conservation. (**C**) Location of modified residues in the NP structure. Red atom stick indicates the location on α-helix regions, green atom stick indicates the location on loop regions. N, N-terminal domain; C, C-terminal domain.

**NP**, an RNA-binding protein that plays a critical role in IAV RNA transcription and replication, was found to be methylated and acetylated, predominantly on arginine and serine, respectively (Table 1). Methylation was detected on R150, R246, R317, K325, R416, and R422, whereas acetylation occurred on S274, S283, S287, S326, S403, and K325. Like in M1, one residue, K325 was found to be either methylated or acetylated. The alignment of 1,124 NP polypeptide sequences from 42 IAV subtypes (Supplementary Table S2) revealed that four of the methylated residues, R150, R246, R317, and R416, and three of the acetylated residues, S274, S287, and S326, as well as dual-modified K325, were highly conserved (Figure 2A). Out of these, R150, R416, and K325 were also conserved within NP of IAV, IBV and ICV. Whereas R246, R317, S326, and S403 were conserved within the NP of IAV and IBV (Figure 2B). Next, we located the modified residues in the NP structure, which is composed of multiple α-helices, β-strands and loop regions [38]. We found that methylated R150, R246, R317, and R416 and acetylated S274 and S403 were all located in the loop regions (Figure 2C). The rest of the modified residues were situated on α-helices: S283 and S287 on α12 helix, K325 and S326 on α15 helix, and R422 on α18 helix (Figure 2C). Evidently, R150, K325, and R416 are indispensable for IAV growth. The R150 and K325 are critical for NP to bind the viral RNA [39] and incorporate it into virus particles [40], respectively; whereas, R416 is important for NP to oligomerise [41]. In addition, R422 also contributes to the NP oligomerisation [42] as well as NP’s interaction with host heterogeneous nuclear ribonucleoprotein (hnRNP) A2/B1, which is involved in IAV RNA synthesis [43]. Markedly, we also found several hnRNPs being modified in response to the IAV infection (described below).

**Figure 3:**
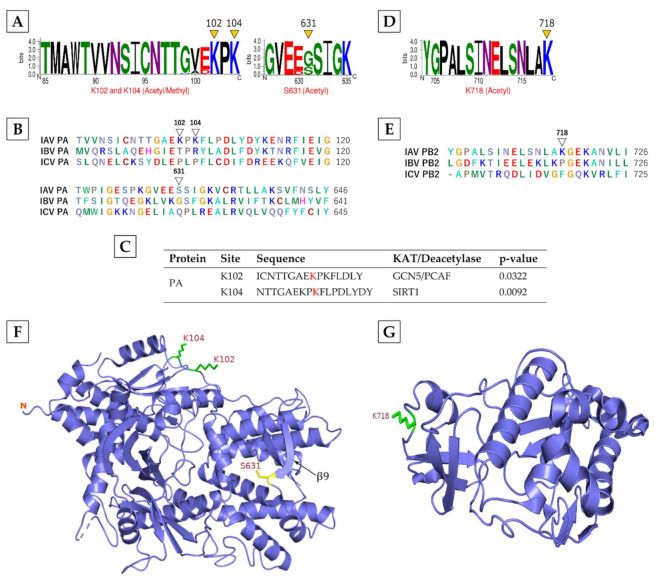
The characteristics of the modifications on IAV PA and PB2. Conservation of modified residues in PA (**A**) and PB2 (**D**) across IAV subtypes. A total of 800 PA and 986 PB2 sequences from 48 different IAV subtype strains were aligned in BioEdit using ClustalW and visualised by WebLogo. The logos represent individual peptides with modified residues (yellow arrowheads). The *X*-axis indicates the position of amino acid and the *Y*-axis indicates the bit score on a scale of 1–4 (A scale of 4 means 100% conservation). Acetyl: acetylation, Methyl: methylation. Conservation of modified residues in PA (**B**) and PB2 (**E**) across IAV, IBV, and ICV. White arrowheads point to no conservation. (**C**) Prediction of identified acetylated sites (in red) to be acetylated or deacetylated by ASEB web software. Location of modified residues in the PA (**F**) and PB2 C-terminal domain (**G**) structures. The green atom stick indicates the location on loop regions, yellow atom stick indicates the location on β-strand. N, N-terminal domain.

**PA, PB1,** and **PB2,** the three IAV RNA polymerase subunits, were also discovered to be modified (Table 1). PA was methylated as well as acetylated on both K102 and K104, and acetylated on S631. In contrast, PB1 and PB2 were N-terminally acetylated and allysine (on K718), respectively. In PA, differentially modified K102 and K104 were highly conserved (Figure 3A) across 800 sequences from 48 IAV subtypes (Supplementary Table S2), though these residues were not conserved within PA of IAV, IBV or ICV (Figure 3B). Furthermore, ASEB analysis predicted that K102 can be acetylated by acetyltransferases GCN5/PCAF and K104 can be deacetylated by SIRT1 (Figure 3C). In PB2, allysine K718 was also highly conserved (Figure 3D) across 986 sequences from 48 IAV subtypes (Supplementary Table S2), but not within the PB2 of IAV, IBV or ICV (Figure 3E). In the PA structure, K102 and K104 were located in the loop region of its critical N-terminal endonuclease domain (Figure 3F), which also binds PB2 [44]. Furthermore, K102 is vital for PA endonuclease activity, particularly for its mRNA cap-binding activity [45] and consequently for IAV growth [46]. The S631 was located on the β9 strand (Figure 3F) within the PB1-binding site of the PA C-terminal domain [47]. In the PB2 structure, K718 was situated in the loop region of the C-terminal “DPDE” domain (Figure 3G). This domain contains a nuclear localisation signal (NLS) and binds to the host nuclear import protein, importin α to facilitate PB2 nuclear import [48].

**Figure 4:**
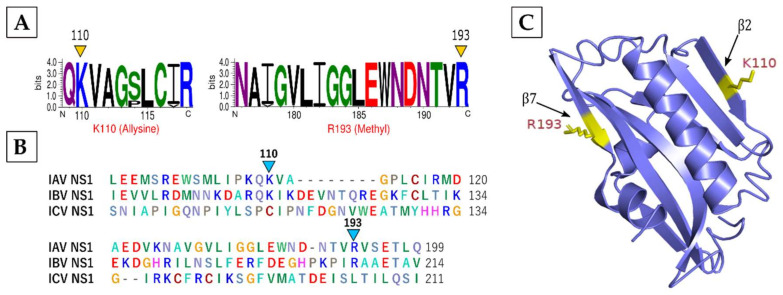
The characteristics of the modifications on IAV NS1. (**A**) Conservation of modified residues across NS1 of IAV subtypes. A total of 998 sequences from 48 different IAV subtype strains were aligned in BioEdit using ClustalW and visualized by WebLogo. The logos represent individual peptides with modified residues (yellow arrowheads). The *X*-axis indicates the position of amino acid and the *Y*-axis indicates the bit score on a scale of 1–4 (A scale of 4 means 100% conservation). Methyl: methylation. (**B**) Conservation of modified residues across NS1 of IAV, IBV, and ICV. Turquoise arrowheads point to conservation in IAV and IBV. (**C**) Location of modified residues in the NS1 effector domain structure. Yellow atom stick indicates the location on β-strands.

**NS1** and **NS2,** two main IAV non-structural proteins were found to be acetylated at the N-terminus (Table 1). Additionally, NS1, which is the main interferon antagonist, was also methylated on R193 and modified by allysine on K110. Both K110 and R193 were highly conserved (Figure 4A) across 998 NS1 sequences belonging to 48 IAV subtypes (Supplementary Table S2). Furthermore, they were also conserved within the NS1 of IAV and IBV (Figure 4B). In the NS1 structure, both K110 and R193 were part of the critical NS1 effector domain [49] and located on β-strands: K110 on β2-strand and R193 on β7-strand (Figure 4C).

**Figure 5:**
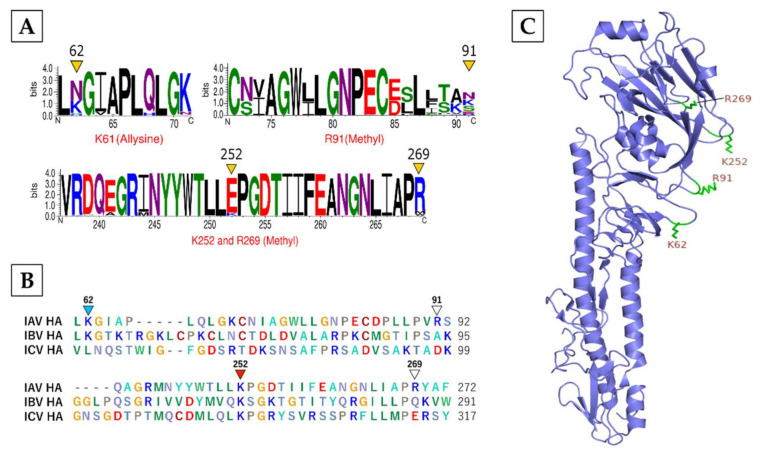
The characteristics of the modifications on IAV hemagglutinin (HA). (**A**) Conservation of modified residues across HA of IAV H1 subtypes. A total of 480 sequences from 7 different IAV H1 subtype strains were aligned in BioEdit using ClustalW and visualized by WebLogo. The logos represent individual peptides with modified residues (yellow arrowheads). The *X*-axis indicates the position of amino acid and the *Y*-axis indicates the bit score on a scale of 1–4 (A scale of 4 means 100% conservation). Methyl: methylation. (**B**) Conservation of modified residues across HA of IAV, IBV, and ICV. Red arrowheads point to conservation in all three influenza virus types, turquoise arrowheads point to conservation in IAV and IBV, and white arrowheads point to no conservation. (**C**) Location of modified residues in the HA structure. Green atom stick indicates the location on loop regions.

**HA**, the receptor-binding protein of IAV that is critical for virus entry, was found to be modified by methylation on R91, K252, and R269 and allysine on K62 (Table 1). HA is the most rapidly evolving protein of IAV. Therefore, the conservation analysis was performed using 480 sequences from only H1 subtypes (Supplementary Table S2). We found that R269 was largely conserved within HA of IAV subtypes (Figure 5A). However, K252 was highly conserved across HA of IAV, IBV, and ICV (Figure 5B). The structural analysis revealed that all modified residues were part of the HA1 globular subunit and located in its loop regions (Figure 5C). As HA1 is further divided into fusion, vestigial esterase and receptor-binding domains [50], the methylated R91 and R269 were situated in the vestigial esterase domain and K252 in the receptor-binding domain, whereas the allysine K62 was situated in the fusion domain (Figure 5C).

### 3.2. Two Hundred and Forty-Five Host Proteins Were Modified at 300 Sites by Methylation, Acetylation, and/or Allysine in Response to IAV Infection

Our proteomic screen identified a total of 2280 host proteins (Supplementary Table S3), out of which, 245 were discovered to be selectively modified by methylation, acetylation and/or allysine in IAV-infected cells. However, the total number of modified sites was 300 because, like viral proteins, some host proteins were modified: (1) by the same modification on multiple residues, (2) by more than one modification, or (3) on the same residues by different modifications (Supplementary Table S4). In addition, 18 proteins were discovered to be modified in both uninfected and infected cells and 54 proteins were selectively modified only in uninfected cells. Since our aim here was to identify the host proteins that were selectively modified in response to IAV infection, these proteins were excluded from further analysis.

**Methylated proteins.** A total of 116 host proteins were found to be uniquely methylated at 159 sites (95 lysine and 64 arginine) in IAV-infected cells (Supplementary Table S4). The majority, 106 of 116 proteins were mono-methylated, but 10 were di-methylated and 1 was tri-methylated. Further, 82 proteins were methylated on one residue and 34 on more than one residue. Out of 34, 28 proteins were methylated on two residues, 4 on three residues (alpha-actinin 1, filamin A and heat shock protein [HSP70]), 1 on four residues (splicing factor 3B subunit 2 [SF3B2]), and 1 on five residues (alpha-enolase 1). Furthermore, 16 of 34 proteins were methylated on both lysine and arginine residues (Supplementary Table S4).

**Figure 6:**
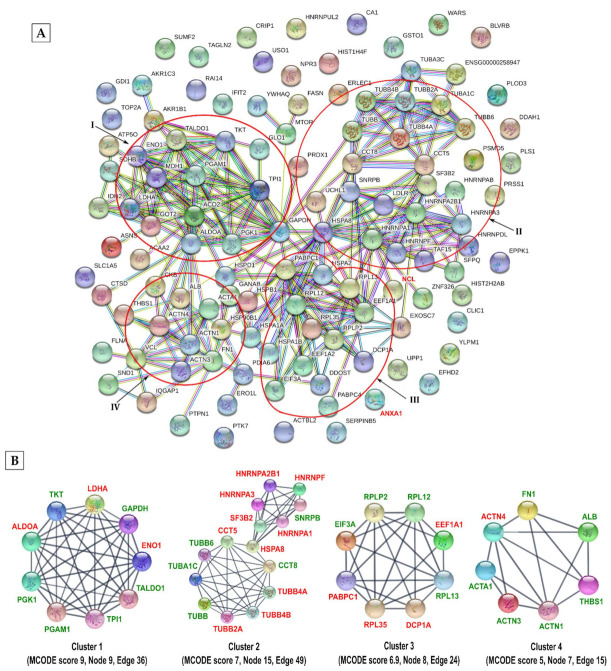
STRING functional protein association network of identified methylated host proteins. (**A**) The identified methylated host proteins from infected cells were analysed for functional protein associations using the STRING database. The network contains 336 edges and 102 nodes with an average node degree of 6.59. The minimum required interaction score was set to high confidence (≥0.700) and PPI enrichment *p*-value was <1.0 × 10^−16^. Major clusters are encircled in red. (**B**) The four most highly ranked and tightly connected methylated protein clusters were obtained by the MCODE algorithm using Cytoscape (version 3.8.0) and ranked based on their MCODE scores. The proteins shown in red are known to be involved in influenza virus infection.

To understand the significance of identified methylated host proteins in IAV infection, we used the STRING database to construct their functional association networks. STRING database processed 102 out of 116 input proteins and constructed a network of 336 interactions between 80 methylated proteins (Figure 6A). The functional enrichment analysis revealed that identified methylated proteins were involved in the positive regulation of viral genome replication (GO:0045070) (FDR = 0.0097). The Cytoscape analysis (with built-in MCODE algorithm) identified four top-ranked interconnected clusters of methylated host proteins (Figure 6B). Cluster 1 (MCODE score: 9) contained enzymes from the glycolytic pathway, namely α-enolase (ENO1; methylated on K80, K221, K228, R269, and K358), fructose-bisphosphate aldolase A (ALDOA; methylated on K384), glyceraldehyde-3-phosphate dehydrogenase (GAPDH; methylated on K55), lactate dehydrogenase A (LDHA; methylated on K110 and K155), phosphoglycerate kinase 1 (PGK1; methylated on K291), phosphoglycerate mutase 1 (PGAM1; methylated on K157), transaldolase (TALDO1; methylated on K81), transketolase (TKT; methylated on K102) and triosephosphate isomerase 1 (TPI1; methylated on K106 and K256) (Supplementary Table S4). Most of the methylated lysine/arginine residues in these enzymes were conserved across the natural (human, pig, chicken, dog, and horse) and experimental (mouse) hosts of IAV (Supplementary Table S5). Evidently, IAV exploits the glycolytic pathway to proliferate as the glucose uptake and metabolism are increased during infection [51,52]. Furthermore, α-enolase (and another glycolytic enzyme, pyruvate kinase M found to be acetylated on K549, see below) is known to interact with IAV M1 protein [53]. Cluster 2 (MCODE score: 7) contained two interconnected mini clusters of 9 and 6 proteins. One mini-cluster comprised of 6 tubulins and 3 chaperones, T-complex protein 1 subunit epsilon, T-complex protein 1 subunit theta, and heat shock cognate 71 (HSPA8) protein, all of which are associated with the intracellular transport. Another mini-cluster contained 5 nuclear ribonucleoproteins and splicing factor SF3B2; all these proteins have been implicated in the RNA splicing. Notably, tubulins through their polymerised form, microtubules facilitate intracellular trafficking of different IAV components [54] and nuclear ribonucleoproteins regulate IAV mRNA splicing and transport [54,55,56,57]. Further, almost all methylated lysine or arginine residues in these proteins were conserved in the above six IAV hosts (Supplementary Table S5). Cluster 3 (MCODE score: 6.9) contained 8 proteins involved in translation (60S ribosomal proteins, elongation factors, and polyadenylate-binding protein 1) and RNA processing (mRNA-decapping enzyme 1A), which were methylated on highly conserved residues (Supplementary Tables S4 and S5). Lastly, cluster 4 (MCODE score: 5) was made up of mainly actin and actin-binding proteins, actinin and filamin. The methylated residues of α-actin (R256), α-acitinin 1 (K633, K682 and K684), and filamin A (K900, R1532 and K2133) were highly conserved across the six IAV hosts (Supplementary Tables S4 and S5). Like microtubules, actin filaments play a critical role during the IAV life cycle, from entry to egress [54,58,59] (Figure 6B). In addition to these clusters, four enzymes of the tricarboxylic acid cycle (TCA cycle), namely aconitate hydratase (K138, K245), isocitrate dehydrogenase (K272), malate dehydrogenase (K238, R248), succinate dehydrogenase (K80) were found to be methylated on largely conserved residues (Supplementary Tables S4 and S5). Evidently, a reduced TCA cycle activity has been reported in IAV-infected mice [60].

Individual host proteins discovered to be methylated here (Supplementary Tables S4 and S5) have also been implicated in IAV infection. For instance, annexin A1, methylated at conserved K26 protects mice from IAV infection [61] and nucleolin, methylated at conserved K333 plays both proviral and antiviral roles during IAV infection [62,63,64,65,66] (Supplementary Tables S4 and S5).

**Acetylated proteins.** We identified 98 host proteins to be uniquely acetylated at 108 sites in infected cells (Supplementary Table S4). Out of these, 20 proteins were acetylated on 24 lysine and 27 on 31 serine residues, and 53 proteins were acetylated at the N-terminus. Most of them were acetylated on one lysine or serine residue; only 8 were acetylated on two lysine or serine residues. Further, as mentioned above, some of the acetylated proteins were also methylated or allysine (Supplementary Table S4).

**Figure 7:**
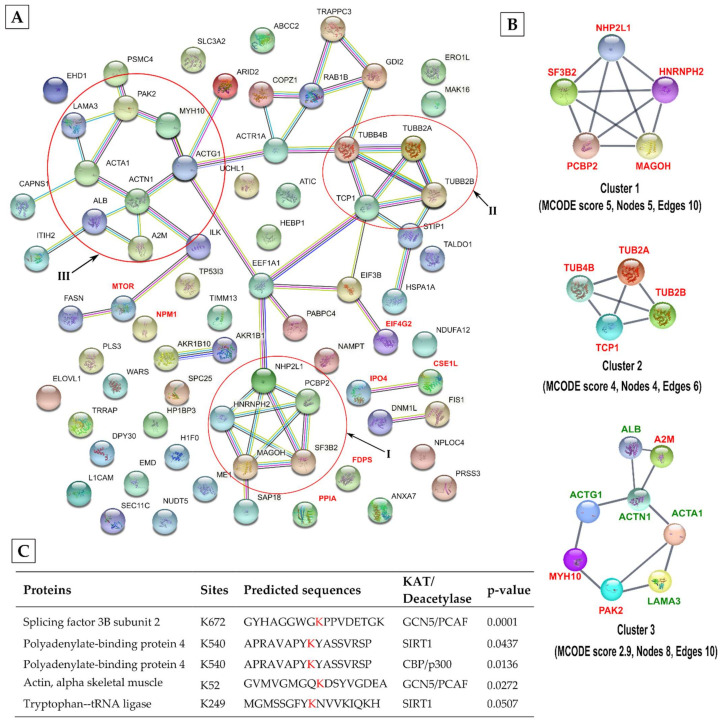
STRING functional protein association network of acetylated host proteins. (**A**) The identified acetylated host proteins from infected cells were analysed for functional protein association using the STRING database. Proteins are shown as nodes while the interactions are shown as edges. The association network contains 63 edges and 77 nodes with an average node degree of 1.64. The minimum required interaction score was set to high confidence (≥0.700) and PPI enrichment *p*-value was2.67 × 10^−5^. Major clusters are encircled in red. (**B**) The three most highly ranked and tightly connected acetylated protein clusters were obtained by the MCODE algorithm using Cytoscape and ranked based on their MCODE scores. The proteins shown in red are known to be involved in influenza virus infection. (**C**) Prediction of identified acetylated sites (in red) to be acetylated or deacetylated by ASEB web software.

All 98 proteins that were either N-terminally acetylated or acetylated on lysine and serine residues were included in functional association networks analyses using the STRING database (Figure 7A). This analysis processed 77 of the acetylated proteins and identified their involvement in viral processes (GO = 0016032) (FDR = 0.0201) and IRES-dependent viral translation initiation (GO = 0075522) (FDR = 0.0289). The MCODE algorithm identified three major interconnected clusters of identified acetylated proteins. The highest-ranked cluster 1 (MCODE score: 5) included SF3B2, poly (rC) binding protein 2 (PCBP2), NHP2L1 and hnRNPH2 (Figure 7B), all of which have been implicated in mRNA splicing. The SF3B2 was acetylated on conserved K672 and K680, and PCBP2 was acetylated on conserved S84, whereas the hnRNPH2 was acetylated at the N-terminus (Supplementary Tables S4 and S5). As mentioned above, IAV utilises the host mRNA splicing machinery, particularly to process its M and NS gene transcripts to produce multiple proteins [67]. Further, PCBP2, which regulates antiviral response, has been identified as the target of miRNA-like small RNA encoded by IAV H5N1 subtype [68]. Cluster 2 (MCODE score: 4) contained 3 tubulins, all acetylated on S364 and associated chaperone T-complex protein 1, acetylated at N-terminus (Figure 7B) (Supplementary Table S4). Cluster 3 included actin, acetylated on highly conserved K52 and S54 and actin-binding proteins: actinin, acetylated on highly conserved K682 and K684; laminin, acetylated on conserved S2164; and associated kinase serine/threonine-protein kinase PAK2, acetylated on S42 (Figure 7B; Supplementary Tables S4 and S5). The significance of the proteins in both clusters 2 and 3 in IAV infection has been described above and elsewhere [69]. In support of this data, ASEB analysis predicted that K672 of SF3B2 and K52 of actin can be acetylated by GCN5/PCAF acetyltransferase (Figure 7C). In addition, ASEB analysis also predicted that the conserved K540 of polyadenylate-binding protein 4 that was identified to be acetylated here, can be acetylated and deacetylated by CBP/p300 and SIRT1, respectively (Figure 7C).

Individual acetylated proteins (Supplementary Tables S4 and S5) not included in any cluster have also been implicated in IAV infection. Farnesyl pyrophosphate synthase (FDPS), acetylated on S73, is the target of anti-IAV host factor viperin and is a pro-IAV factor [70]. Serine/threonine-protein kinase mTOR, acetylated on conserved K1068, is a component of mammalian target of rapamycin (mTOR) complex and known to promote IAV infection [71]. The N-terminally acetylated eukaryotic translation initiation factor 4GI (eIF4GI) is recruited by IAV NS1 for preferential translation of viral proteins [49] and nucleophosmin interacts with viral ribonucleoprotein (vRNP—a complex of IAV NP, RNA, and RNA polymerase subunits PA, PB1, PB2) and contributes to viral RNA synthesis [72]. Peptidyl-prolyl cis-trans isomerase A or cyclophilin A (PPIA), acetylated at the N-terminus, restricts IAV infection by targeting M1 [73]. Finally, importin 4 (IPO4) and exportin 2 (CSE1L), both acetylated at the N-terminus, are the members of host nuclear transport machinery which is critical for IAV replication [1].

**Allysine proteins.** In response to IAV infection, 29 host proteins were found to be selectively modified by allysine on 33 lysine residues (Supplementary Table S4). Of these, only 2, guanine nucleotide-binding protein G(i) subunit and HSPA8 were modified on two (K9, K14) and four (K25, K159, K328, K384) lysine residues, respectively (Supplementary Tables S4 and S5). Further, as mentioned above, some allysine proteins were also methylated or acetylated (Supplementary Table S4). Importantly, all lysine residues identified to be modified by allysine were unique and not found to be methylated or acetylated.

**Figure 8:**
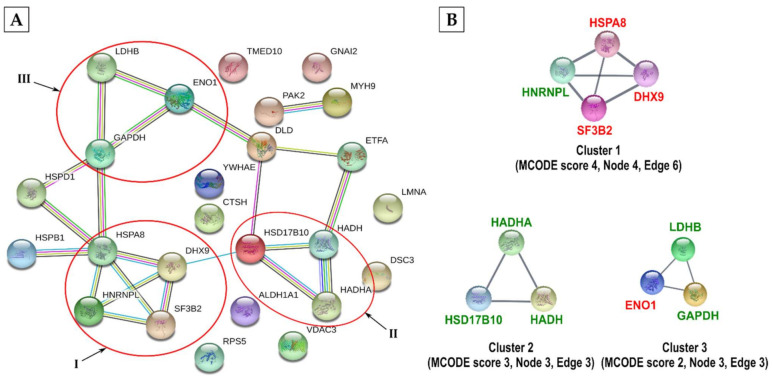
STRING functional protein association network of allysine host proteins. (**A**) The identified allysine host proteins from infected cells were analysed for functional protein association using the STRING database. Proteins are shown as nodes while interactions are shown as edges. The association network contains 22 edges and 25 nodes with an average node degree of 1.76. The minimum required interaction score was set to high confidence (≥0.700) and PPI enrichment *p*-value was <3.2 × 10^−6^. Major clusters are encircled in red. (**B**) The three most highly ranked and tightly connected protein clusters were obtained by the MCODE algorithm using Cytoscape. The clusters were ranked based on their MCODE scores. The proteins shown in red are known to be involved in influenza virus infection.

Out of 29 proteins, 25 were processed by the STRING database and 22 interactions were detected amongst them (Figure 8A). They were further grouped into three main clusters by the MCODE algorithm. Cluster 1 with an MCODE score of 4 contained four RNA binding and processing proteins: hnRNP L (allysine on highly conserved K178), SF3B2 (allysine on K697), and ATP-dependent RNA helicase A (DHX9; allysine on K314) and associated chaperone HSPA8 (allysine on highly conserved K25, K159, K328, and K384) (Figure 8B) (Supplementary Tables S4 and S5). Cluster 2 contained fatty acid metabolism enzymes, 3-hydroxyacyl-CoA dehydrogenase type-2 and hydroxyacyl-coenzyme A dehydrogenase that were allysine on highly conserved K172 and K178 residues, respectively (Figure 8B) (Supplementary Tables S4 and S5). Finally, cluster 3, like cluster 1 of methylated proteins (Figure 6B), is comprised of three glycolytic enzymes, ENO1, GAPDH and LDH B allysine on K326, K9 and K14 and highly conserved K229, respectively (Figure 8B) (Figure 8B) (Supplementary Tables S4 and S5). Serine/threonine-protein kinase PAK2, which was part of acetylated cluster 3 above (Figure 7B), was also allysine on K38 (Supplementary Table S4).

## 4. Discussion

The role and significance of lysine acetylation and lysine/arginine methylation have been extensively studied in histones modifications and their influence on gene expression. However, these modifications are now known to occur in many non-histone proteins and contribute to, for example, their subcellular localisation and organelle retention (N-terminal acetylation) [2], intracellular transport (lysine acetylation) [2], and role in RNA processes such as splicing (arginine methylation) [3]. Evidently, both viral and host proteins with these characteristics are involved in IAV infection [1], and, in this study, we have identified such proteins with those modifications. Further, the identified host proteins were distributed across intracellular compartments and the plasma membrane of the host cell (Supplementary Figure S2).

M1 and NP proteins carried the most modifications of all IAV proteins, with multiple modifications on the same and different amino acid residues. M1 and NP perform multiple functions during the IAV life cycle, from viral entry to RNA synthesis to assembly. To execute these functions, they shuttle between the cytoplasm and nucleus and to the plasma membrane and interact with various host as well as viral proteins and RNAs [74]. Potentially, the modifications on M1 and NP identified herein are crucial to conduct those functions. In M1, K95 was methylated as well as acetylated and K98 was methylated as well as allysine. Both these residues are located on M1 “helix six”, a region of positively-charged amino acids containing an NLS and responsible for multiple M1 functions, including binding to vRNP and lipid membrane [36]. Therefore, it is conceivable that different modifications on K95 and K98 dynamically influence the interactions of M1 with vRNP and lipid membrane. The NP, an RNA-binding protein, was methylated mostly on arginine residues (R150, R246, R317, R416 and R422). Evidently, the arginine methylation of RNA-binding proteins such as hnRNPs—several of which have also been found here to be selectively methylated on arginine in infected cells, is common and regulates their nucleocytoplasmic shuttling and role in transcription and mRNA splicing [75,76]. Therefore, methylation of R150, which resides in the NP groove region that binds viral RNA [39], may influence NP’s role in viral transcription and vRNP nuclear import/export. Furthermore, methylation of K317 and R416 may contribute to NP-NP oligomerisation by governing the salt bridge formation with residue E369 [77] and E339 [78], respectively. Evidently, methylation of lysine influences the conformational changes in HSP90 through salt bridge formation [79]. In addition, NP was also acetylated on multiple serine residues (S274, S283, S287, S326, and S403), of these S274 is part of the NP nuclear export signal 3 (NES3) which facilitates the export of vRNP from the nucleus to the cytoplasm [74,80]. The role of serine acetylation in nuclear transport is yet to be identified, but lysine acetylation does regulate nuclear export [81,82]. Furthermore, N-terminal acetylation of the nuclear transport proteins, exportin 2 and importin 4 in infected cells detected here, may also regulate vRNP nuclear transport.

Likewise, identified modifications of other IAV proteins also potentially influence their already known functions. For example, methylation and acetylation of viral RNA polymerase subunit PA on both K102 and K104 in the endonuclease domain potentially regulate PA binding to viral RNA [45]. Indeed, methylation as well as acetylation of HIV-1 Tat on two adjacent lysine residues (K50 and K51) is known to regulate its binding to RNA [83]. The allysine of PB2 on K718 in the NLS domain may determine its diverse binding activity to NLS-binding pockets of importin α isoforms [84]. Similarly, allysine and methylation of NS1 in the effector domain (on K110 and R193, respectively) potentially regulate its binding to host cleavage and polyadenylation specificity factor 30 (CPSF30) and poly(A)-binding protein II (PABII) and facilitate the sequestering of host mRNAs. Furthermore, these modifications may also facilitate the interaction of NS1 with eIF4GI for preferential translation of IAV proteins [49]. Incidentally, eIF4GI was also found to be N-terminally acetylated in the infected cells. Finally, the methylation of HA on R269, which is part of a crucial “tetrad” salt bridge, potentially influences its irreversible conformational change at acidic pH in endosomes during IAV entry [85].

On host proteins, methylation was the most abundant modification followed by acetylation and allysine in infected cells. Nevertheless, the methylated, acetylated, and allysine host proteins in infected cells were commonly annotated to three protein-protein interaction clusters implicated in metabolic (glycolysis, TCA cycle, lipid metabolism), cytoskeletal, and RNA processes. Evidently, these three processes are known to be involved in IAV infection and their proteins interact with viral proteins in various stages of the IAV life cycle [51,54,67,86]. Further, the identified modifications have been known to influence the function of some of those proteins. For example, methylation of GAPDH is associated with its enhanced catalytic activity in pancreatic cancer [87]; acetylation alters the activity and stability of pyruvate kinase M in cancer [88]; acetylation regulates the stability of actin filaments and microtubules, hence intracellular transport [2], including of the IAV components [18]; and, finally, methylation regulates the mRNA splicing [75,76].

In conclusion, our data point to strong alterations in the landscape of methylation, acetylation and allysine of both viral and host proteins in response to IAV infection. The modifications in viral proteins occurred on conserved residues in critical domains, whereas the modified host proteins belonged to important cellular pathways that are known to be exploited by IAV. Furthermore, SIRT1 and GCN5/PCAF (predicted to deacetylate and acetylate, respectively, some acetylated viral and host proteins identified here) have been described to play an antiviral and proviral role, respectively, during influenza virus infection [19,89]. Therefore, the significance of these modifications on some viral and host proteins vis-à-vis IAV infection is evidently clear, but on others, it seems to be rather complex. Experimental validation of the functional significance of the identified modifications, on both conserved and non-conserved residues will shed further light on it. This study had some limitations. We were unable to detect known lysine acetylation sites that were previously reported to be present on viral NP and NS1 [20,21,90]. However, these reports used selective samples that were enriched either by immunoprecipitation, ectopic overexpression, or ultracentrifugation to detect the lysine acetylation sites. In our view, the enrichment and targeted sample strategies are narrow and selective and will confound the detection of other modifications. Our goal was to detect the modifications that enriched and occurred specifically in response to IAV infection and to gain insights into possible crosstalk between different modifications. Most of the modifications are kinetically dynamic in response to a stimulus like infection, and the turnover of lysine acetylation is faster than methylation [91]. For similar reasons, the mutagenesis of modified residues will unlikely reveal the significance of a modification on a protein, because the same residue can undergo dynamic and alternative multiple modifications as we have seen here. The alternative experimental approaches [5,92] that navigate these challenges (using a model of primary human respiratory cells/tissues) will need to be applied in tandem to reveal and validate the full extent of global protein modifications and their significance during IAV infection.

## Figures and Tables

**Table 1:** Identified methylated, acetylated, and allysine sites in major influenza A virus (IAV) proteins.

Viral Protein	Modification	Modified Peptide
**M1**	Methyl [K95] *	[R].FVQNALNGNGDPNNMDK.[A]
	Methyl [K98] *	[R].FVQNALNGNGDPNNMDKAVK.[L]
	Methyl [R160]	[R].MGAVTTEVAFGLVCATCEQIADSQHR.[S]
	Methyl [K230] *	[R].TIGTHPSSSAGLKNDLLENLQAYQK.[R]
	Methyl [K242]	[K].NDLLENLQAYQK.[R]
	Acetyl [S195]	[K].AMEQMAGSSEQAAEAMEVASQAR.[Q]
	Acetyl [S196]	[K].AMEQMAGSSEQAAEAMEVASQAR.[Q]
	Acetyl [S207]	[K].AMEQMAGSSEQAAEAMEVASQAR.[Q]
	Acetyl [K95] *	[R].FVQNALNGNGDPNNMDK.[A]
	Allysine [K35]	[R].LEDVFAGKNTDLEVLMEWLK.[T]
	Allysine [K98] *	[R].FVQNALNGNGDPNNMDKAVK.[L]
	Allysine [K230] *	[R].TIGTHPSSSAGLKNDLLENLQAYQK.[R]
**NP**	Methyl [R150]	[R].QANNGDDATAGLTHMMIWHSNLNDATYQR.[T]
	Methyl [R246]	[R].ESRNPGNAEFEDLTFLAR.[S]
	Methyl [R317]	[R].LLQNSQVYSLIRPNENPAHK.[S]
	Methyl [K325] *	[R].LLQNSQVYSLIRPNENPAHK.[S]
	Methyl [R416]	[R].ASAGQISIQPTFSVQR.[N]
	Methyl [R422]	[R].NLPFDRTTVMAAFTGNTEGR.[T]
	Acetyl [S274]	[K].SCLPACVYGSAVASGYDFER.[E]
	Acetyl [S283]	[K].SCLPACVYGSAVASGYDFER.[E]
	Acetyl [S287]	[K].SCLPACVYGSAVASGYDFER.[E]
	Acetyl [S326]	[K].SQLVWMACHSAAFEDLR.[V]
	Acetyl [S403]	[R].ASSGQISIQPTFSVQR.[N]
	Acetyl [K325] *	[R].LLQNSQVYSLIRPNENPAHK.[S]
**PA**	Methyl [K102] *	[R].TMAWTVVNSICNTTGAEKPK.[F]
	Methyl [K104] *	[R].TMAWTVVNSICNTTGAEKPK.[F]
	Acetyl [K102] *	[R].TMAWTVVNSICNTTGAEKPK.[F]
	Acetyl [K104] *	[R].TMAWTVVNSICNTTGAEKPK.[F]
	Acetyl [S631]	[K].GVEESSIGK.[V]
**PB1**	N-term acetyl	[-].MDVNPTLLFLK.[V]
**PB2**	Allysine [K718]	[R].YGPALSINELSNLAK.[G]
**NS1**	Methyl [R193]	[K].NAVGVLIGGLEWNDNTVR.[V]
	Allysine [K110]	[K].QKVAGPLCIR.[M]
	N-term acetyl	[-].MDPNTVSSFQVDCFLWHVR.[K]
**NS2**	N-term acetyl	[-].MDPNTVSSFQDILLR.[M]
**HA**	Methyl [R91]	[K].CNIAGWLLGNPECDLLLPVR.[S]
	Methyl [R269]	[K].VRDQAGRMNYYWTLLKPGDTIIFEANGNLVAPR.[Y]
	Methyl [K252]	[R].MNYYWTLLKPGDTIIFEANGNLIAPR.[Y]
	Allysine [K62]	[R].LKGITPLQLGK.[C]

Red indicates modified amino acids; * indicates amino acids with more than one modification.

## Data Availability

The data presented in this study are available as supplementary material or from the authors upon request.

## Supplementary Materials

The following are available online at https://www.mdpi.com/article/10.3390/v13071415/s1. Figure S1: Influenza A virus (IAV) titer and polypeptide profile of infected cells used for mass spectrometry, Figure S2: Subcellular localization of the identified modified host proteins based on their GO term, Table S1: Detected IAV proteins and their modifications, Table S2: Number of viral protein sequences and IAV subtypes used for alignments, Table S3: List of total number of identified host proteins, Table S4: List of total number of identified host proteins undergoing the modifications and their modified sites, Table S5: Conservation of the modified sites in host proteins across human, pig, chicken, dog, horse, and mouse.
