# Supplementary material for: Acetylation, Methylation and Allysine Modification Profile of Viral and Host Proteins during Influenza A Virus Infection
Source: Viruses. 2021 Jul 20;13(7):1415. doi: 10.3390/v13071415 (PMC8310381; doi:10.3390/v13071415)
Supplement: Supplementary file 1 [file viruses-13-01415-s001.zip › viruses-1281647-supplementary/viruses-1281647 Supplementary Figure S1,S2 Table S1,S2.pdf]

viruses

*Supplementary Information*

# Acetylation, Methylation and Allysine Modification Profile of Viral and Host Proteins during Influenza A Virus Infection

**Farjana Ahmed [1], Torsten Kleffmann [2] and Matloob Husain [1,*]**

[1] Department of Microbiology and Immunology, University of Otago, P.O. Box 56, Dunedin 9054, New Zealand; farjana.ahmed@postgrad.otago.ac.nz
[2] Centre for Protein Research, Research Infrastructure Centre, University of Otago, P.O. Box 56, Dunedin 9054, New Zealand; torsten.kleffmann@otago.ac.nz
* Correspondence: matloob.husain@otago.ac.nz

**Citation:** Ahmed, F.; Kleffmann, T.; Husain, M. Acetylation, Methylation and Allysine Modification Profile of Viral and Host Proteins During Influenza A Virus Infection. *Viruses* **2021**, *13*, 1415. https://doi.org/10.3390/v13071415

Academic Editor: Craig McCormick

Received: 16 June 2021
Accepted: 18 July 2021
Published: 20 July 2021

**Publisher's Note:** MDPI stays neutral with regard to jurisdictional claims in published maps and institutional affiliations.

**Copyright:** © 2021 by the authors. Licensee MDPI, Basel, Switzerland. This article is an open access article distributed under the terms and conditions of the Creative Commons Attribution (CC BY) license (http://creativecommons.org/licenses/by/4.0/).

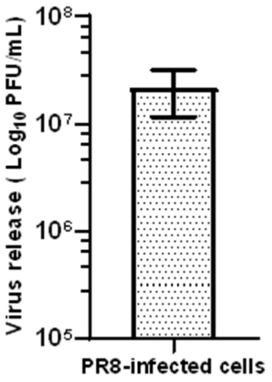


(**A**)

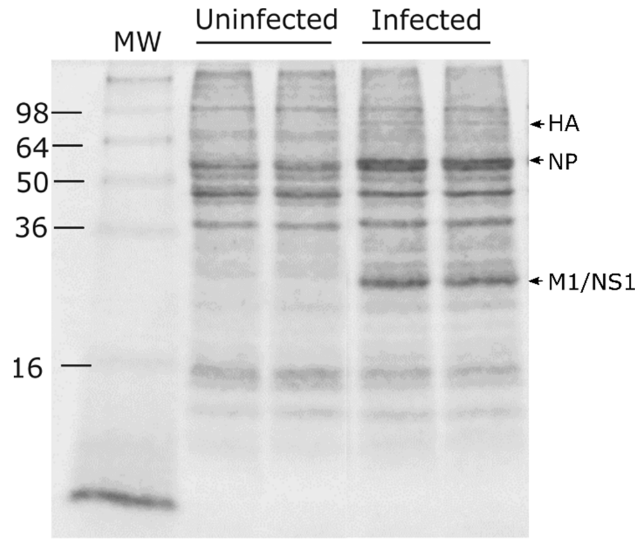


(**B**)

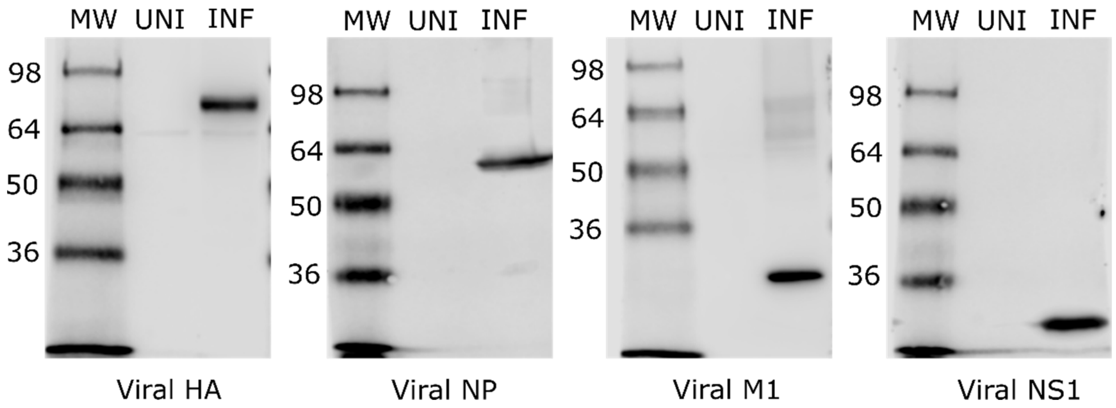


(**C**)

**Figure S1.** Influenza A virus (IAV) titer and polypeptide profile of infected cells used for mass spectrometry**.** A549 cells were grown in 25 cm$^2$ cell culture flasks to 95% confluency and infected with IAV at MOI of 1.0 for 24 hours. The cells and the media were harvested separately. The media was titrated on MDCK cells by plaque assay to determine the titer of released viral progeny (**A**). Whereas the cells were lysed, and total cell lysates were resolved on 15% SDS-PAGE (**B**). The gel was stained with Coomassie blue and imaged on Odyssey Fc imager (Li-COR). (**C**) Total cell lysates from uninfected (UNI) and infected (INF) A549 cells were resolved on 10% SDS-PAGE, and viral HA (80 kDa), NP (56 kDa), M1 (28 kDa), and NS1 (26 kDa) polypeptides were detected by western blotting using the method described elsewhere (ref. 15). The antibodies to HA (NR-3148), NP (NR-19868), and NS1 (NR-44426) were obtained through BEI Resources (NIAID, NIH, USA) and antibody to M1 (G122) was kindly provided by Richard Webby (St Jude Children's Research Hospital, USA). MW, molecular weight in kDa.

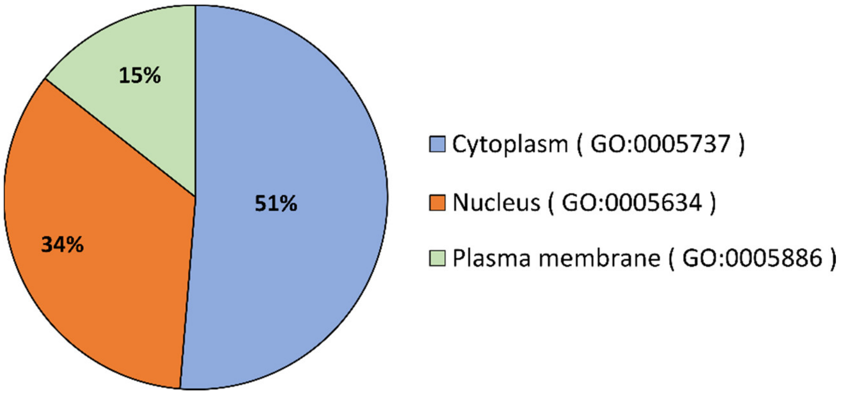


**Figure S2.** Subcellular localization of the identified modified host proteins based on their GO term.

**Table S1.** Detected IAV proteins and their modifications.

| No | Accession | Protein description | Coverage [%] | #Peptides | #PSMs | #Unique Peptides | MW [kDa] | Score SEQUEST HT | Modifications | XCorr |
|---|---|---|---|---|---|---|---|---|---|---|
| 1 | ABP64734.1 | Matrix protein 1 [Influenza virus A/PR/8/34(H1N1)] | 89 | 34 | 2806 | 34 | 27.9 | 7192.23 | Methyl [K95] | 4.36 |
| | | | | | | | | | Methyl [K98] | 3.81 |
| | | | | | | | | | Methyl [R160] | 4.30 |
| | | | | | | | | | Methyl [K230] | 3.82 |
| | | | | | | | | | Methyl [K242] | 2.03 |
| | | | | | | | | | Acetyl [S195] | 4.61 |
| | | | | | | | | | Acetyl [S196] | 3.85 |
| | | | | | | | | | Acetyl [S207] | 3.68 |
| | | | | | | | | | Acetyl [K95] | 4.15 |
| | | | | | | | | | Allysine [K35] | 2.71 |
| | | | | | | | | | Allysine [K98] | 2.20 |
| | | | | | | | | | Allysine [K230] | 4.15 |
| 2 | AAM75162.1 | Matrix protein M2 [Influenza virus A/PR/8/34/Mt Sinai(H1N1)] | 30 | 2 | 86 | 2 | 11 | 217.39 | -- | - |
| 3 | NP_040982.1 | Nucleoprotein [Influenza virus A/PR/8/1934(H1N1)] | 79 | 49 | 4167 | 6 | 56.1 | 10707.22 | Methyl [R150] | 2.44 |
| | | | | | | | | | Methyl [R246] | 4.20 |
| | | | | | | | | | Methyl [R317] | 2.56 |
| | | | | | | | | | Methyl [K325] | 2.56 |
| | | | | | | | | | Methyl [R416] | 2.27 |
| | | | | | | | | | Methyl [R422] | 4.44 |
| | | | | | | | | | Acetyl [S274] | 5.18 |
| | | | | | | | | | Acetyl [S283] | 6.23 |
| | | | | | | | | | Acetyl [S287] | 4.50 |
| | | | | | | | | | Acetyl [S326] | 3.73 |
| | | | | | | | | | Acetyl [S403] | 2.95 |
| | | | | | | | | | Acetyl [K325] | 2.86 |
| 4 | ABP64720.1 | Polymerase acidic protein [Influenza virus A/hvPR8/34(H1N1)] | 49 | 33 | 410 | 33 | 82.5 | 957.94 | Methyl [K102] | 2.85 |
| | | | | | | | | | Methyl [K104] | 2.51 |
| | | | | | | | | | Acetyl [K102] | 3.15 |
| | | | | | | | | | Acetyl [K104] | 2.30 |
| | | | | | | | | | Acetyl [S631] | 1.91 |
| 5 | ABO21706.1 | Polymerase basic protein 1 [Influenza virus A/PR/8/1934(H1N1)] | 40 | 28 | 357 | 28 | 86.5 | 921.28 | Acetyl [N-Term] | 3.10 |
| 6 | ABP64718.1 | Polymerase basic protein 2 [Influenza virus A/hvPR8/34(H1N1)] | 52 | 39 | 523 | 39 | 86.1 | 1513.78 | Allysine [K718] | 4.02 |
| 7 | NP_040984.1 | Non-structural protein 1 [Influenza virus A/PR/8/1934(H1N1)] | 68 | 20 | 1401 | 20 | 25.9 | 3886.3 | Methyl [R193] | 3.31 |
| | | | | | | | | | Allysine [K110] | 2.26 |
| | | | | | | | | | Acetyl [N-Term] | 4.03 |

| No | Accession | Protein description | Coverage [%] | #Peptides | #PSMs | #Unique Peptides | MW [kDa] | Score SE-QUEST HT | Modifications | XCorr |
|---|---|---|---|---|---|---|---|---|---|---|
| 8 | ABP64727.1 | Non-structural protein 2 [Influenza virus A/hvPR8/34(H1N1)] | 65 | 7 | 89 | 7 | 14.4 | 236.82 | Acetyl [N-Term] | 2.28 |
| 9 | CAA24272.1 | Haemagglutinin [Influenza virus (A/PR/8/1934(H1N1))] | 54 | 31 | 1908 | 4 | 63.3 | 4720.2 | Methyl [R91] | 1.82 |
| | | | | | | | | | Methyl [R269] | 3.52 |
| | | | | | | | | | Methyl [K252] | 3.53 |
| | | | | | | | | | Allysine [K62] | 3.41 |
| 10 | ABP64723.1 | Neuraminidase [Influenza virus (A/hvPR8/34(H1N1)] | 35 | 18 | 550 | 18 | 50.1 | 1672.05 | -- | - |

Coverage: The percentage of full protein sequence that is covered by the identified peptides, #Peptides: The number of distinct peptide sequences in a protein group, #PSMs (Peptide Spectra Matches): The total number of identified peptide sequences for the target protein, #Unique peptides: The number of peptide sequences unique to a protein group, Score SEQUEST HT: The sum of the individual scores of each peptide, Xcorr (cross correlation): The number of fragment ions that are common to two different peptides with same precursor mass.

**Table S2.** Number of viral protein sequences and IAV subtypes used for alignments.

| IAV Proteins | Number of Sequences | IAV Subtypes |
|---|---|---|
| M1 | 800 | H1N1, H1N2, H1N3, H2N2, H2N3, H2N5, H2N9, H3N2, H3N6, H3N8, H4N5, H4N6, H4N7, H4N9, H5N1, H5N2, H5N3, H5N8, H5N9, H6N1, H6N2, H6N3, H6N5, H6N6, H6N8, H7N1, H7N2, H7N3, H7N7, H9N2, H9N9, H10N3, H10N4, H10N7, H11N7, H11N8, H11N9, H12N5, H13N6 |
| NP | 1124 | H1N1, H1N2, H1N9, H2N2, H2N3, H2N5, H2N9, H3N2, H3N6, H3N8, H4N2, H4N5, H4N6, H4N9, H5N1, H5N2, H5N3, H5N8, H5N9, H6N1, H6N2, H6N3, H6N5, H6N6, H6N8, H7N1, H7N3, H7N7, H7N8, H7N9, H8N4, H9N2, H9N9, H10N3, H10N4, H10N7, H11N7, H11N9, H12N2, H12N4, H12N5, H14N6 |
| PA | 800 | H1N1, H1N2, H1N3, H1N9, H2N2, H2N3, H2N5, H2N9, H3N2, H3N6, H3N8, H4N2, H4N6, H4N5, H4N7, H4N9, H5N1, H5N2, H5N3, H5N8, H5N9, H6N1, H6N2, H6N5, H6N8, H7N1, H7N3, H7N7, H7N8, H7N9, H8N4, H9N2, H9N9, H10N3, H10N4, H10N5, H10N6, H10N7, H10N8, H11N2, H11N7, H11N8, H11N9, H12N2, H12N4, H12N5, H13N9, H14N6 |
| PB2 | 986 | H1N1, H1N2, H1N3, H2N2, H2N3, H2N5, H2N9, H3N2, H3N6, H3N8, H4N2, H4N6, H4N5, H4N7, H4N9, H5N1, H5N2, H5N3, H5N8, H5N9, H6N1, H6N2, H6N4, H6N5, H6N8, H7N1, H7N3, H7N7, H7N8, H7N9, H8N4, H9N2, H9N9, H10N3, H10N4, H10N5, H10N6, H10N7, H10N8, H11N2, H11N7, H11N8, H11N9, H12N2, H12N4, H12N5, H13N9, H14N6 |
| NS1 | 998 | H1N1, H1N2, H1N3, H2N2, H2N3, H2N5, H2N9, H3N2, H3N6, H3N8, H4N2, H4N6, H4N5, H4N7, H4N9, H5N1, H5N2, H5N3, H5N8, H5N9, H6N1, H6N2, H6N5, H6N8, H7N1, H7N3, H7N7, H7N8, H7N9, H8N4, H9N2, H9N9, H10N3, H10N4, H10N5, H10N6, H10N7, H10N8, H11N2, H11N7, H11N8, H11N9, H12N2, H12N4, H12N5, H13N6, H13N9, H14N6 |
| HA | 480 | H1N1, H1N2, H1N3, H1N5, H1N6, H1N7, H1N9 |
